# Influence of Nb Addition on the Microstructure and Mechanical Properties of Laser Powder Bed Fusion-Fabricated Ti6Al4V Alloy

**DOI:** 10.3390/ma18081803

**Published:** 2025-04-15

**Authors:** Bo Zhang, Min Wan, Na Gao, Hao Jiang, Tuokuan Chen, Peng Zhao, Zhenzhen Liu, Qingyuan Jiao, Baoguo Lv, Quanquan Han, Xiebin Wang

**Affiliations:** 1 Shandong Institute of Medical Device and Pharmaceutical Packaging Inspection, Jinan 250101, China; 2 School of Materials Science and Engineering, Shandong University, Jinan 250061, Chinaxiebin.wang@email.sdu.edu.cn (X.W.); 3 School of Mechanical Engineering, Shandong University, Jinan 250061, China; 4 Department of Mechanical Engineering, Lunan Technical College, Linyi 276017, China

**Keywords:** additive manufacturing, laser powder bed fusion, titanium alloy, Ti6Al4V, Nb addition, in situ alloying

## Abstract

Additive manufacturing of Ti6Al4V alloys via laser powder bed fusion (L-PBF) has demonstrated superior tensile strength compared to conventional methods. However, challenges remain in enhancing ductility and tailoring mechanical properties for specific applications. In this work, we show a feasible method to regulate the mechanical properties of additively manufacturing Ti alloys. Ti6Al4V alloys with different Nb content (1, 3, and 10 wt.%) were fabricated through laser powder bed fusion (L-PBF) in situ alloying using the mixture of Ti6Al4V and Nb powders. The powder mixture shows good printability, and dense Ti6Al4V-xNb alloys are obtained. Although the distribution of Nb is highly heterogeneous, no solidification cracks or secondary intermetallics were detected in both the Nb-rich and Nb-lean regions. The microstructure is gradually refined with the increase in Nb addition, mainly due to the heterogeneous nucleation caused by the partially melted Nb particles. The L-PBF-fabricated T6Al4V-xNb alloys are mainly in α’ martensite phase, even with the addition of 10 wt.% Nb, due to the low content of Nb solute in the matrix. The presence of β phase is suggested around the Nb particles, since a small region with graded Nb content is formed around the Nb particles. The ultimate tensile strength increases from 1050 to 1181 MPa with the addition of 3 wt.% Nb, and the total elongation increases slightly from 8.8% to 10.5%. With the addition of 10 wt.% Nb, the total elongation increases largely to 15.6%, while maintaining a high strength of 1135 MPa. Moreover, the elastic modulus decreases from 105 to 80 GPa with the increase in Nb content to 10 wt.%. The results of this work suggest that L-PBF in situ alloying is a promising approach to optimize the mechanical performance of Ti6Al4V alloys.

## 1. Introduction

Titanium and its alloys show excellent mechanical properties, promising strength-to-weight ratio, exceptional corrosion resistance, and good biocompatibility, which have been widely used in many fields, such as aerospace parts, marine components, and medical implants [1]. Ti6Al4V is classified as an α + β titanium alloy, where α phase has a hexagonal close-packed (HCP) crystal structure, and β phase has a body-centered cubic (BCC) crystal structure. The displacive transformation from β phase to the hexagonal martensite α’ phase may also occur upon rapid cooling [1,2]. Ti6Al4V is one of the most commercially used Ti alloys, especially for biomedical applications [3,4,5,6]. Nevertheless, the broad applications of Ti alloys are largely limited, due to the difficulties in machining the Ti alloy parts, since Ti alloys generally show poor thermal conductivity, low elastic modulus, and high reactivity [7,8,9].

Additive manufacturing (AM) technology, which builds complex metallic components through a layer-wise process, is an ideal method to address the challenges in producing geometrically complex metallic parts [10,11,12]. Ti6Al4V represents one of the pioneering Ti alloys to be successfully implemented for AM, especially for laser powder bed fusion (L-PBF) [13,14,15,16]. Many studies have shown that large columnar prior β, which grows across several layers along the build direction, is first developed at elevated temperature (above the β-transus temperature) in L-PBF-fabricated Ti6Al4V alloy [14,17]. Afterwards, the displacive β → α’ transformation occurs due to the extremely high cooling rate associated with the L-PBF process, leading to the formation of acicular α’ martensite inside the prior β grains [14,18,19]. The unique microstructure results in the high strength of the L-PBF-fabricated Ti6Al4V alloy, but the ductility is low (generally < 10%) [17,19,20].

Heat treatment is an efficient method to optimize the microstructure and mechanical properties of L-PBF-fabricated Ti6Al4V alloys [15,21,22,23,24,25,26]. The acicular α’ martensite decomposes gradually into α and β phase upon heating, and the fraction of the two phases can be tailored by modifying the heat treatment parameters (e.g., temperature, holding time) [15,19,25,26]. The formation of the β phase and thickening of the α plates lead to a decrease in the strengths, but the ductility could be largely improved to well above > 10%. For instance, Vrancken et al. [19] reported that the fracture strain increases from ~5% to ~14% with the increase in annealing temperature from 540 °C to 1020 °C. Post-heat treatment also helps largely to improve the fatigue performance of the L-PBF-fabricated Ti6Al4V parts. Recently, Qu et al. [27] proposed a Net-AM process, i.e., hot isostatic processing (HIP), followed by solution treatment at 1050 °C for 4 min and aging at 500 °C for 6 h, which improves significantly the fatigue resistance to a fatigue limit of around 1GPa.

Apart from producing the complex Ti alloy structures, L-PBF can also act as a metallurgical method to improve the mechanical performance of Ti alloys, when using powder mixture as feedstocks [7,28,29,30,31]. Zhang et al. [30] reported that the strength and ductility of the L-PBF-fabricated Ti5553 alloy could be improved by adding 5 wt.% Mo, due to the refinement of the microstructure caused by the partially melted Mo particles. Vrancken et al. [31] obtained full β phase in L-PBF-fabricated Ti6Al4V alloy by adding 10 wt.% Mo, which lowers largely the β-transus temperature. An ultrafine-grained Ti-Cu alloy, which shows high strength and uniform elongation, is developed by Zhang et al. [28] through L-PBF in situ alloying using the Ti and Cu powder mixture.

Nb is frequently used as a β stabilizer in Ti-based alloys, and many Ti-Nb-based alloys have been developed [1,32], e.g., Ti-6Al-7nb [33], Ti-24Nb-4Zr-8Sn [34], and Ti-25Nb-2Mo-4Sn [35]. Ti alloys could transfer from α type to α + β type, and eventually β type alloy with the increase in Nb content [1]. The appearance of the α’, α”, and ω phases were also reported, depending on the Nb content and thermal history [36]. The introduction of ductile β phase and Nb can improve the ductility of Ti alloys. Moreover, the addition of Nb could reduce the elastic modulus of Ti alloys, which is attractive for biomedical applications.

In order to improve the ductility, and the combination of strength and ductility of the L-PBF-fabricated Ti6Al4V alloy, this work presents an attempt to explore a feasible method to optimize the mechanical performance of the Ti6Al4V alloy through L-PBF in situ alloying using a mixture of Ti6Al4V and elemental Nb powders. The influence of Nb addition on the microstructure and mechanical properties of L-PBF-fabricated Ti6Al4V alloy was studied. A high strength of 1142 MPa and good ductility (total elongation of >15%) are obtained in L-PBF-fabricated Ti6Al4V alloy by adding 10 wt.% Nb.

## 2. Materials and Methods

**Figure 1:**
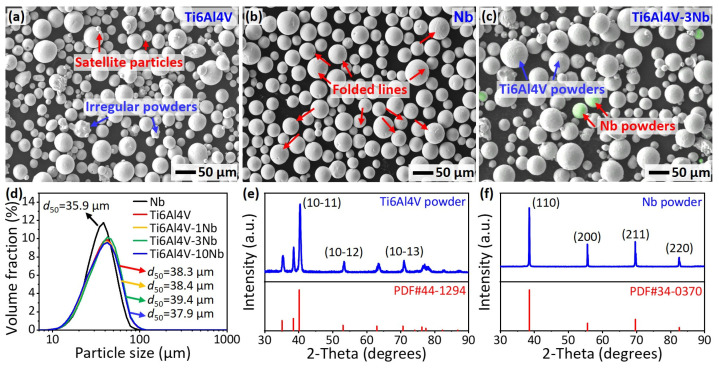
Secondary electron images of (**a**) Ti6Al4V, (**b**) Nb, and (**c**) Ti6Al4V-3Nb powders. The Nb powders are visualized by overlapping the energy-dispersive X-ray spectroscopy (EDS) scans. (**d**) Particle size distribution of Ti6Al4V, Nb, and Ti6Al4V-xNb (x = 1, 3, 10 wt.%) powders. (**e**) and (**f**) show the X-ray diffraction profiles of the Ti6Al4V and Nb powders, respectively.

In this work, spherical Ti6Al4V (Avimetal AM Tech. Co., Ltd., Beijing, China) and Nb (Chengfeng Materials Technology Co., Ltd., Foshan, China) powders were used. The Ti6Al4V alloy powders were fabricated using the electrode induction gas atomization (EIGA) technology. Most of the powders are spherical, but some irregular powders and satellite particles are readily seen, as shown in Figure 1a. The Nb powders were produced using the plasma spheroidization (PS) technology [37]. Figure 1b shows the morphology of the Nb powders. As compared with the Ti6Al4V powders, the Nb powders are more uniform and have less irregular and satellite particles. The folded lines present on the surface of the Nb powders, which is typical for PS-made powders [38,39].

The Ti6Al4V-Nb powder mixtures with Nb content of 1, 3, 10 wt.%, labeled as Ti6Al4V-1Nb, Ti6Al4V-3Nb, and Ti6Al4V-10Nb, respectively, were prepared through mechanically mixing the Ti6Al4V and Nb powders in a tumbler mixer (WAB T2G, Muttenz, Switzerland) for 8 h. Figure 1c shows the morphology and distribution of the Ti6Al4V-3Nb powders. The Nb powders are visualized by overlapping the EDS (energy-dispersive X-ray spectroscopy) scans. Figure 1c indicates a uniform distribution of Nb particles in the powder mixture. Figure 1d provides the particle size distribution of the powders. Both the Ti6Al4V and Nb powders show sizes between 10 and 100 μm, with *d_50_* of 38.3 and 35.9 μm, respectively. The addition of Nb powders has less influence on the particle size distribution of the powder mixture, due to the small Nb content in the powder mixture. Figure 1e,f show the X-ray diffraction profiles, collected at room temperature, of the Ti6Al4V and Nb powders, respectively. The Ti6Al4V powders are mainly in α phase, and Nb powders are mainly in the BCC phase.

**Figure 2:**
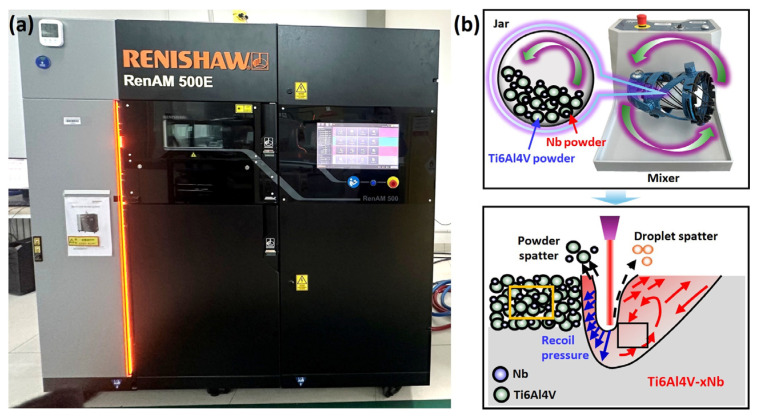
(**a**) The laser powder bed fusion system (Renishaw RenAM 500E) used in this work. (**b**) Illustration on the L-PBF in situ alloying method of this work.

**Figure 3:**
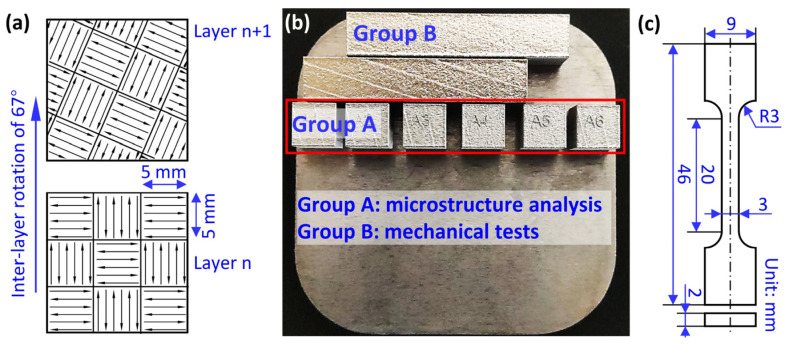
(**a**) Schematic plot of the chessboard scanning strategy. (**b**) A photo of the as-printed samples. (**c**) The dimension of the specimens for tensile tests.

All the samples were fabricated using a commercial L-PBF machine (Figure 2a, Renishaw RenAM 500E, Wotton-under-Edge, UK), which is equipped with a 500 W fiber laser. Figure 2b illustrates the methodological framework of this work. The optimized L-PBF process parameters are laser power (*P*) of 160 W, laser scanning speed (*v*) of 1100 mm/s, hatch spacing (*h*) of 80 μm, and layer thickness (*t*) of 30 μm. The chessboard scanning strategy, with an island size of 5 × 5 mm^2^ and interlayer rotation angle of 67°, was employed (Figure 3a). In order to minimize the contamination from oxygen, pure argon (purity of >99.999%) was used to protect the printing process. As shown in Figure 3b, the cubic samples (Group A) with size of 10 × 10 × 10 mm^3^ were fabricated for characterizing the microstructure. The cuboid samples (Group B) with a dimension of 60 × 10 × 10 mm^3^ were produced for tensile tests. The dog-bone-shaped specimens have a gauge length of 15 mm, width of 3 mm, and thickness of 2 mm (Figure 3c). The thick direction is parallel to the build direction.

The surface of the sample was mechanically grinded and polished for characterizing the microstructure. A ZEISS G500 scanning electron microscope (SEM, Oberkochen, Germany) equipped with an Ultim Max (Oxford Instruments, Abingdon, UK) energy-dispersive X-ray spectroscopy (EDS) detector was used to analyze the microstructure. The elemental distribution was investigated using an electron probe microanalyzer (EPMA, JEOL JXA-8530F Plus, Tokyo, Japan). The electron backscatter diffraction (EBSD) technique was employed to study the grain morphology and crystallographic information. The EBSD measurements were conducted using a JEOL JSM-7800 SEM equipped with a NordlysMax3 (Oxford Instruments, Abingdon, UK) detector. The phase composition at room temperature was analyzed using a Rigaku MiniFlex X-ray diffractometer (XRD, Tokyo, Japan) with Cu Kα radiation. Unidirectional tensile tests were performed using an MTS universal test machine (mode E44.304, Eden Prairie, MN, USA) under a constant deformation rate of 2%/min. The tensile strain was recorded using an extensometer.

## 3. Results and Discussion

### 3.1. Microstructure and Elemental Distribution

**Figure 4:**
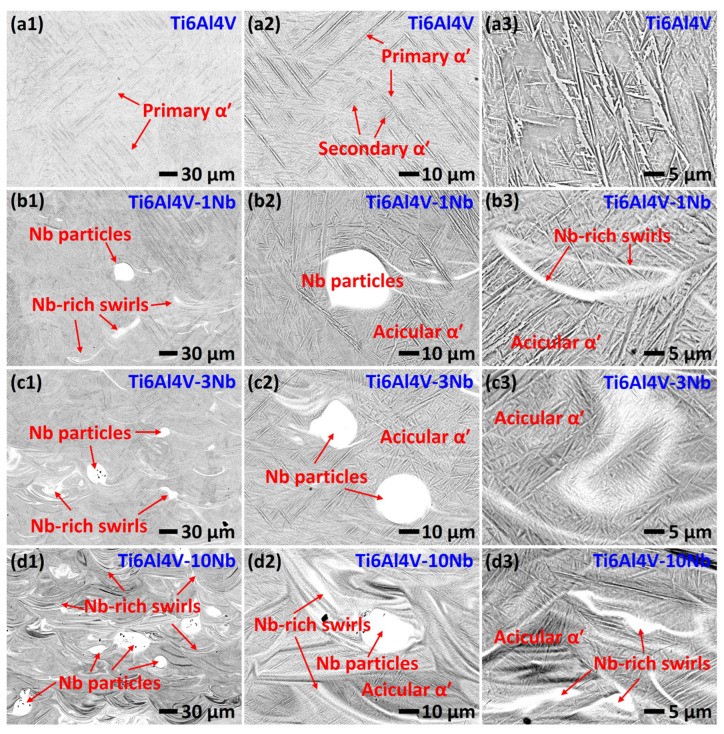
The SEM-BSE images of the L-PBF-fabricated (**a1**–**a3**) Ti6Al4V, (**b1**–**b3**) Ti6Al4V-1Nb, (**c1**–**c3**) Ti6Al4V-3Nb, (**d1**–**d3**) Ti6Al4V-10Nb alloys. The observation surface is parallel to the build direction.

Figure 4 shows the backscattered electron (BSE) images of the L-PBF-fabricated Ti6Al4V alloy with different Nb additions. The observation surface is parallel to the build direction. The as-printed Ti6Al4V alloy is composed of fully acicular α’ martensite phase (Figure 4(a1–a3)), which originates from the extremely high cooling rate associated with the L-PBF process (10^6^–10^8^ °C/s [40,41,42]). The rapid cooling suppresses largely the diffusive β → α and promotes the displacive β → α’ transition [1,2]. Figure 4(a1–a3) shows that the α’ phase, with a basket-weave pattern, consists of hierarchical acicular martensite, including (i) primary α’ martensite, which has a high aspect ratio and length of up to 200 µm; (ii) secondary α’ martensite, which grows in between the primary α’ laths. The finer tertiary and quartic α’ martensite have also been reported in previous studies [43], while further studies need to be conducted to investigate the finer α’ martensite. The hierarchical acicular α’ martensite contributes largely to the high strength of the L-PBF-fabricated Ti6Al4V alloys [14].

Figure 4(b1–b3) shows the BSE images of the L-PBF-fabricated Ti6Al4V-1Nb alloy. Besides the acicular α’ martensite, there are bright regions, which correlate to the partially melted Nb particles and Nb-rich swirls, indicating that the distribution of Nb is not homogeneous. This is mainly due to the large difference in melting point between Nb (2477 °C) and Ti6Al4V (1645 °C [44]) alloys, and the large Nb particles cannot be fully melted. The Nb-rich swirls show the traces of melt-pools, which are driven by Marangoni convection, recoil pressure, and rapid cooling. No crack or secondary particle is observed around the Nb particles, indicating a strong metallurgical bond between the Nb particles and Ti6Al4V matrix. Moreover, unlike previously reported in the NiTi-Nb pseudo-binary system [41,45], dendritic structure is not formed around the Nb particles. This is due to the fact that Ti and Nb can form a continuous solid solution, with Nb and Ti being fully miscible at elevated temperatures, according to the equilibrium Ti-Nb binary phase diagram [46,47,48]. The simple solidification process prevents largely the formation of harmful intermetallic and solidification cracks during the L-PBF process, which helps to maintain the excellent mechanical properties of the Ti6Al4V-*x*Nb alloys.

Figure 4(b2,b3) also show that the partially melted Nb particles and Nb-rich swirls are surrounded with acicular α’ martensite laths. It is known that Nb is a β phase stabilizing element, which will lower the α → β transformation temperature of Ti alloys [1,49,50]. However, a high Nb content (>36 at.% [1]) is required to obtain β phase at room temperature. For instance, Li et al. [51] reported a β-type Ti alloy fabricated by L-PBF with Nb content of 41 wt.%. Therefore, Figure 4(b2,b3) indicate that the content of Nb in the matrix is low, even at the vicinity of the Nb particles, which is due to the limited chemical diffusion under rapid cooling during the L-PBF process.

The BSE images shown in Figure 4(c1–d3) indicate that the microstructure of the Ti6Al4V-3Nb and Ti6Al4V-10Nb is qualitatively similar to the Ti6Al4V-1Nb alloy, except that the number of Nb-rich swirls and partially melted Nb particles is higher with the increase in Nb addition. Figure 4(d3) indicates that the matrix is composted mainly with the acicular α’ martensite in between the Nb-rich swirls. However, the size of the α’ laths are refined as compared with the Ti6Al4V alloy (Figure 4(a3)).

**Figure 5:**
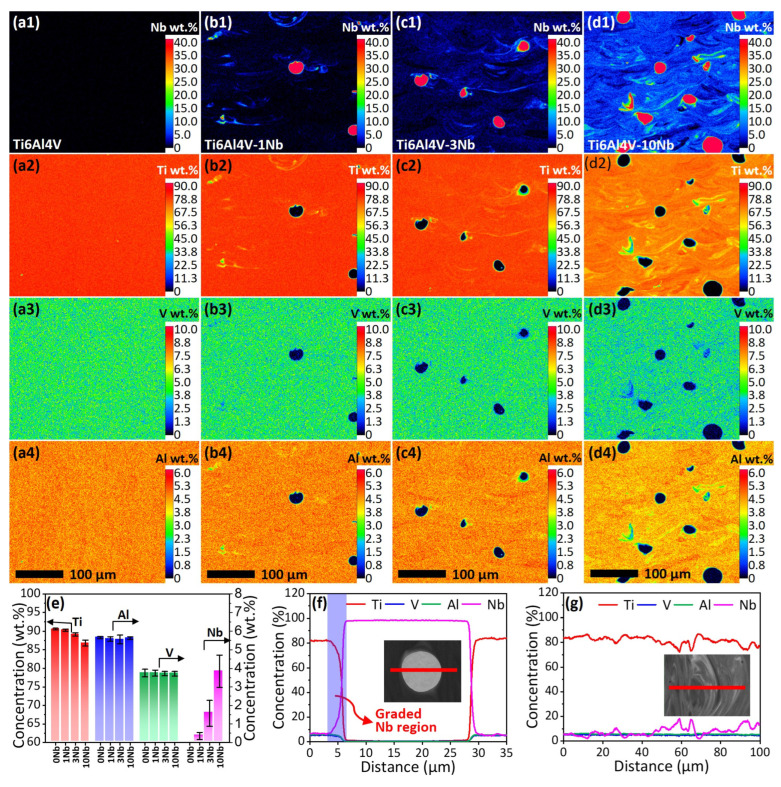
The chemical distribution, as characterized by EPMA, of the L-PBF-fabricated (**a1**–**a4**) Ti6Al4V, (**b1**–**b4**) Ti6Al4V-1Nb, (**c1**–**c4**) Ti6Al4V-3Nb, (**d1**–**d4**) Ti6Al4V-10Nb alloys. (**e**) The content of Ti, Al, V, and Nb in the matrix, which was measured at the place well away from the Nb particles or Nb-rich swirls. The values are an average of 4 measurements. (**f**) and (**g**) are the chemical distribution profiles, measured by EPMA line scan, across the Nb particle and Ni-rich swirls, respectively, in the L-PBF-fabricated Ti6Al4V-3Nb alloy.

Figure 5 provides the distribution of Nb, Ti, V, and Al, as characterized by EPMA, in the L-PBF-fabricated Ti6Al4V-xNb alloys (x = 0, 1, 3, 10 wt.%). A homogeneous distribution of Ti, Al, and V is observed in the L-PBF-fabricated Ti6Al4V alloy (Figure 5(a1–a4)). Figure 5(b1–d4) show that the distribution of Nb is highly heterogeneous, and the number of partially melted Nb particles and Nb-rich swirls increases with the increase in Nb addition from 1 to 10 wt.%. Moreover, the Nb content in the matrix also increases with the Nb addition, indicating the solution of Nb atoms in the Ti6Al4V matrix. Figure 5e provides the Nb content in the matrix, which was measured at the places well away from the Nb particles or Nb-rich swirls. The values in Figure 5e are the average of four measurements. The Nb content in the matrix increases from 0.3 to 3.8 wt.% with the addition of Nb powder from 1 to 10 wt.%. Due to the low content of Nb in the matrix, the L-PBF-fabricated Ti6Al4V-xNb alloys are mainly in α’ phase (Figure 4).

Figure 5f,g present the composition profiles across the partially melted Nb particles and Nb-rich swirls in the L-PBF-fabricated Ti6Al4V-3Nb alloy. Figure 5f indicates the presence of a small area (width of around 3 μm) around the Nb particles, where the Nb content in the matrix increases sharply (the graded Nb region in Figure 5f). It is suggested that β phase may present in the graded Nb region, when the Nb content is higher than 36 wt.% [1]. Figure 5g shows the fluctuation of Nb content across the Nb-rich swirls, but the Nb content is generally below 20 wt.%. This explains the reason that the Nb-rich swirl areas are composed mainly of acicular α’ martensite (Figure 4).

### 3.2. Phase Composition and Crystallography

**Figure 6:**
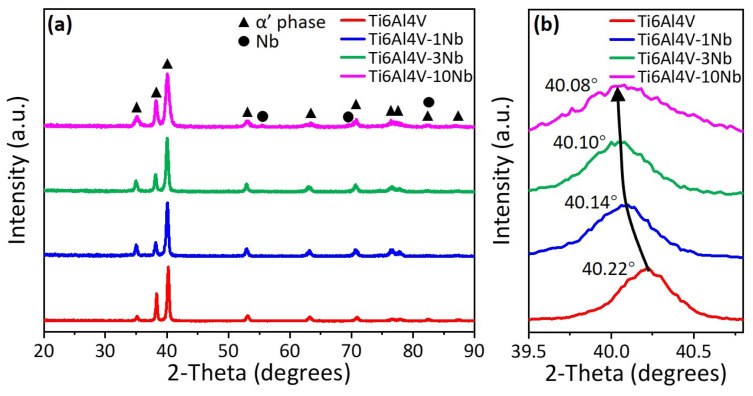
(**a**) The X-ray diffraction profiles of the L-PBF-fabricated Ti6Al4V-xNb (x = 0, 1, 3, 10 wt.%) alloys. (**b**) The shift of (10-11)_α’_ peaks with respect to the increase in Nb content.

Figure 6 shows the phase composition, as characterized by XRD at room temperature, of the L-PBF-fabricated Ti6Al4V-xNb alloys (x = 0, 1, 3, 10 wt.%). All samples are mainly in α’ martensite phase. Only a small diffraction peak related to Nb is observed in the Ti6Al4V-10Nb alloy. The results are consistent with the microstructure observed by SEM (Figure 4). Figure 6b indicates that the position of (10-11)_α’_ peak shifts gradually to the lower 2θ angle side with the increase in Nb addition. The (10-11)_α’_ peak of the as-printed Ti6Al4V is detected at 2θ of 40.22°, while it decreases to 40.08° for the Ti6Al4V-10Nb alloy. This suggests an expansion of the lattice, due to the solution of Nb atoms into the matrix, since Nb has a larger atomic size than Ti, Al, and V.

**Figure 7:**
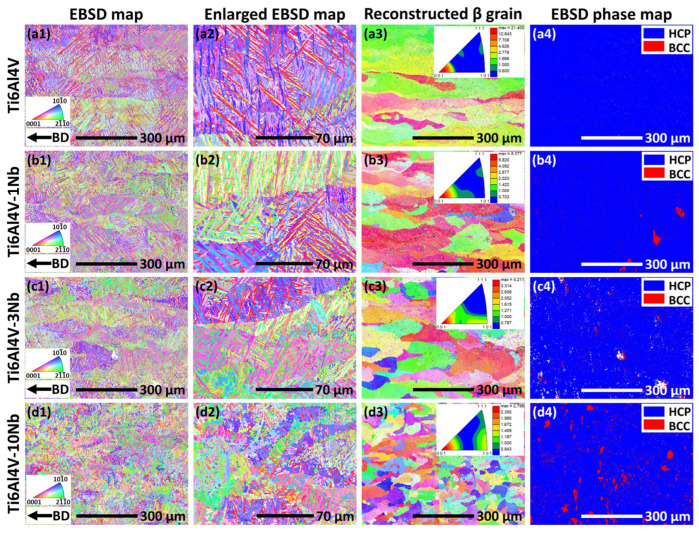
The microstructure, as characterized by EBSD, of the L-PBF-fabricated (**a1**–**a4**) Ti6Al4V, (**b1**–**b4**) Ti6Al4V-1Nb, (**c1**–**c4**) Ti6Al4V-3Nb, (**d1**–**d4**) Ti6Al4V-10Nb alloys. The first column represents the colored crystallographic orientation with reference to the build direction (BD). The second column is the enlarged EBSD map. The third column provides the reconstructed β grains, which was recalculated based on the crystallographic relationship of {110}_β_//{0001}_HCP_ and <1-11>_β_//<11-20>_HCP_. The last column provides the distribution of the BCC (β-Ti and Nb) and HCP (α’) phases.

Figure 7 provides the crystallographic information, as collected by EBSD, of the Ti6Al4V-xNb alloys (x = 0, 1, 3, 10 wt.%). The surface for EBSD observation is parallel to the build direction. The as-printed Ti6Al4V alloy shows mainly the hierarchical acicular α’ martensite phase (Figure 7(a1,a2)). The α’ laths present inside the prior β grains. Figure 7(a3) shows the reconstructed β grains, which was recalculated based on the crystallographic relationship of {110}_β_//{0001}_HCP_ and <1-11>_β_//<11-20>_HCP_ [18,52]. Large columnar grains are observed for the reconstructed β grains, which have been frequently reported in L-PBF-fabricated metallic materials (e.g., Ta [53], Inconel 718 [54], NiTi alloys [55,56]). The formation of the large columnar grains originates from the steep temperature gradient, directional global heat flux, as well as the epitaxial growth of the grains during the L-PBF process [40,57,58]. Moreover, <100> is the easy growth direction for cubic structured crystals [17,59]. As a result, a strong crystallographic texture of <100>//BD is developed in the prior β grains (insert in Figure 7(a3)). Figure 7(a4) provides the phase distribution map of the L-PBF-fabricated Ti6Al4V alloy, which consists of only the HCP structured α’ martensite phase.

Figure 7(b1–b4) presents the EBSD results of the L-PBF-fabricated Ti6Al4V-1Nb alloy. Figure 7(b1,b2) indicate that the microstructure is essentially similar to the Ti6Al4V alloy (Figure 7(a1,a2)), which consists mainly of the hierarchical acicular α’ martensite. Large columnar β grains also developed after 1 wt.% Nb, as indicated in Figure 7(b3). Figure 7(b4) shows that the body-centered cubic (BCC) phase region exists in the Ti6Al4V-1Nb alloy. It is worth mentioning that distinguishing the β-Ti phase and Nb through EBSD technique is rather difficult, since they both have a BCC structure and very similar lattice constant (0.325 nm for β-Ti, and 0.330 nm for Nb). Nevertheless, both the BCC phases are ductile, and will help to improve the ductility of the as-printed samples.

Figure 7(c1–c4) shows the EBSD map of the Ti6Al4V-3Nb alloy. The acicular α’ martensite is also observed, but the sizes of the α’ laths are refined (Figure 7(c1,c2)). Figure 7(c3) also indicates the refinement of the prior β grains after the addition of 3 wt.% Nb, which is due to the fact that the partially melted Nb particles disturb the epitaxial growth of the β grains during L-PBF. Figure 7(c4) shows that the content of the BCC phase increases with the increase in Nb addition, mainly due to the increase in the partially melted Nb particles.

Figure 7(d1,d2) illustrate that the α’ martensite is largely refined after adding 10 wt.% Nb. The refinement of the α’ martensite is due to the decrease in the prior β grain size (Figure 7(d3)), since α’ grows mainly inside the prior β grains. The increase in the partially melted Nb particles disturbs the epitaxial growth and promotes the heterogeneous nucleation during the L-PBF process, leading to the refinement of the prior β grains. The grain refinement also helps to alleviate the strong crystallographic anisotropy of the L-PBF-fabricated metallic materials [59,60]. As illustrated in Figure 7(a3,b3,c3,d3), the maximum intensity of the <100>//BD texture for the prior β grains, based on the inverse pole figures, decreases gradually from 21.4 to 2.8 with the increase in Nb content from 0 to 10 wt.%.

**Figure 8:**
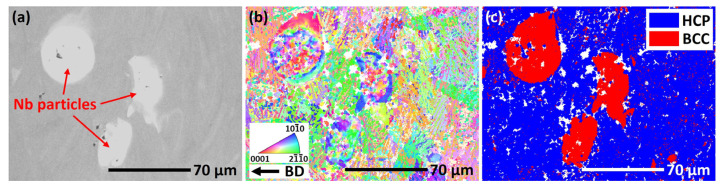
The microstructure, as characterized by EBSD, around the partially melted Nb particles in the L-PBF-fabricated Ti6Al4V-10Nb alloy: (**a**) SEM image, (**b**) colored crystallographic orientation with reference to the build direction, and (**c**) distribution of the BCC and HCP phases.

In order to investigate the phase composition around the partially melted Nb particles, a detailed EBSD scan was performed, as shown in Figure 8. The corresponding SEM image (Figure 8a) indicates the presence of the partially melted Nb particles and Nb-rich swirls. Figure 8b,c show that the matrix is mainly in α’ martensite phase. The BCC phase is mainly the partially melted Nb particles, and most of the Nb-rich swirls are in α’ phase. This is consistent with the XRD results, indicating that the Nb content in the matrix is low.

### 3.3. Mechanical Properties and Fractography

**Figure 9:**
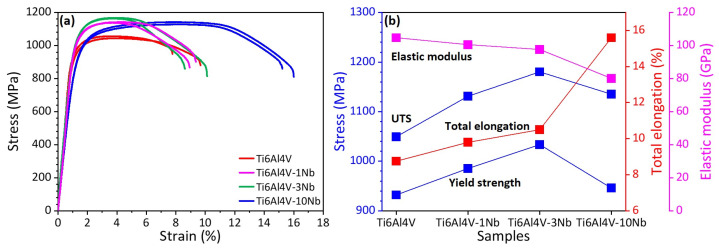
The mechanical properties of the L-PBF-fabricated Ti6Al4V alloy with different Nb content: (**a**) Stress–strain curves, (**b**) summary of the ultimate tensile strength (UTS), yield strength, total elongation, and elastic modulus. The data presented in (**b**) are an average value of the two specimens in (**a**). All the samples were fabricated with *P* = 160 W and *v* = 1100 mm/s.

Figure 9a provides the tensile stress–strain curves of the L-PBF-fabricated Ti6Al4V-xNb alloys (x = 0, 1, 3, 10 wt.%). The yield strength, ultimate tensile strength (UTS), total elongation, and the elastic modulus (E) are summarized in Figure 9b. The yield strength is determined using the 0.2% offset method. The value in Figure 9b is an average value of the two specimens in Figure 9a. The loading direction is perpendicular to the build direction.

As shown in Figure 9, the as-printed Ti6Al4V alloy shows a high strength of around 1050 MPa, and total elongation of 8.7%, which is comparable to the results reported in literature [19,27,31,61]. The high strength originated from the formation of fine hierarchical α’ phase during rapid cooling, which has been frequently discussed in literature [14,19,62,63]. Both the strength and ductility of the Ti6Al4V-xNb alloy increase gradually with the increase in Nb addition from 1 to 3 wt.%. As shown in Figure 9b, the Ti6Al4V-1Nb alloy shows a UTS of 1131 MPa, and further increases to 1181 MPa for the Ti6Al4V-3Nb alloy. The strengthening is caused mainly by the solution of Nb atoms in the Ti matrix. As shown in Figure 4 and Figure 7, both the Ti6Al4V-1Nb and Ti6Al4V-3Nb are composed mainly with α’ phase, which is the main contribution of strength, since the acicular α’ phase martensite provides a strong barrier to the dislocation activities. The extra strengthening caused by the solution of Nb atoms leads to a higher strength of the Ti6Al4V-1Nb and Ti6Al4V-3Nb than the Ti6Al4V alloy. Figure 9b shows that the total elongation of the Ti6Al4V alloy is 8.7%, and it increases slightly to 10.5% after adding 3 wt.% Nb.

The ductility increases largely after adding 10 wt.% Nb. As shown in Figure 9, the Ti6Al4V-10Nb alloy shows a total elongation of 15.6%. As shown in Figure 4, Figure 5, and Figure 7, there are Nb-rich localities around the partially melted Nb particles. As a result, a gradient in Nb content between the Nb particles and Ti6Al4V matrix exists. The high Nb content at certain areas will lead to the appearance of β phase. The soft Nb particles and β phase are the main reason for the high ductility of the Ti6Al4V-10Nb alloy. The Ti6Al4V-10Nb alloy also maintains a high strength of 1142 MPa, indicating that in situ alloying with Nb is an efficient method to improve the performance of L-PBF-fabricated Ti6Al4V alloys.

Figure 9 also indicates that the Ti6Al4V-10Nb alloy shows a low elastic modulus. The E values of the as-printed Ti6Al4V and Ti6Al4V-10Nb alloys are 105 and 80 GPa, respectively. Similar results have been previously reported; for instance, Ehtemam-Haghighi et al. [64] found that the elastic modulus of the Ti-Fe alloy decreases gradually from 129 to 84 GPa with the addition of Nb from 0 to 11 wt.%. This is mainly due to the formation of the β phase. The BCC-structured β phase has a lower atomic packing density than the α and α’ phase, which leads to a lower elastic modulus [64,65]. The materials with low elastic modulus are attractive for orthopedic applications, since the small elastic modulus mismatch between metallic implants and surrounding bone is helpful for alleviating the stress shielding effect [66,67,68].

**Figure 10:**
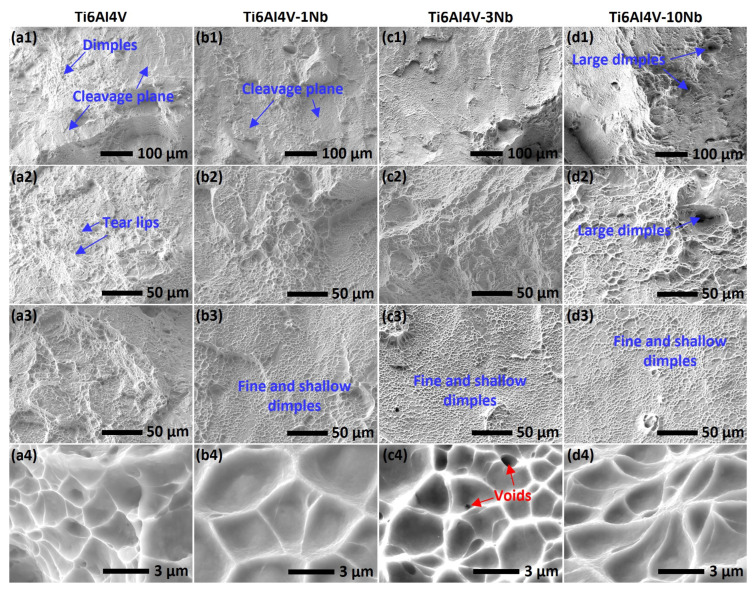
The morphology of the tensile fracture surface of the L-PBF-fabricated (**a1**–**a4**) Ti6Al4V, (**b1**–**b4**) Ti6Al4V-1Nb, (**c1**–**c4**) Ti6Al4V-3Nb, and (**d1**–**d4**) Ti6Al4V-10Nb alloy. All the samples were fabricated with *P* = 160 W and *v* = 1100 mm/s.

Figure 10 shows the fracture surfaces of the as-printed Ti6Al4V alloy with different Nb additions (1, 3 and 10 wt.%) after tensile tests. As shown in Figure 10(a1–a4), the fracture surface of the Ti6Al4V alloy is characterized by the mixture of dimples, tear lips, and cleavage plan, indicating a brittle-ductile mixture fracture. The fracture morphology of the Ti6Al4V-1Nb and Ti6Al4V-3Nb is essentially similar to the Ti6Al4V alloy (Figure 10(b1–c4)). The sheet with fine and shallow dimples is also observed, as shown in Figure 10(b3,c3), indicating the presence of void sheet fracture morphology [69]. In the Ti6Al4V-10Nb alloy, large dimples are frequently observed, probably linked to the Nb-rich localities (Figure 4, Figure 5, and Figure 7), which provide better ductility than the α’ phase.

## 4. Conclusions

In this work, Ti6Al4V alloys with different Nb content (1, 3, and 10 wt.%) were fabricated through L-PBF in situ alloying using the mixture of Ti6Al4V and Nb powders. The influence of Nb addition on the microstructure and mechanical properties of the L-PBF-fabricated Ti6Al4V alloys is studied. The ductility of the L-PBF-fabricated Ti6Al4V alloy is enhanced largely by adding Nb, and an improved balance between strength and ductility is achieved. The main conclusions are summarized as follows:

1. The powder mixture shows good printability. Dense and defect-free Ti6Al4V-xNb alloys are produced successfully. Although the distribution of Nb is highly heterogeneous, no solidification cracks or secondary intermetallics were detected in the Nb-rich areas.
2. The Ti6Al4V-xNb alloys are mainly in α’ martensite phase, even with the addition of 10 wt.% Nb, mainly due to the low content of Nb solute in the matrix. A narrow area with graded Nb content is detected around the partially melted Nb particles, and β phase is suggested to form in this area.
3. Large columnar prior β grains are formed in the L-PBF-fabricated Ti6Al4V alloy. The prior β grain is refined gradually with the increase in Nb addition, since the partially melted Nb particles promote largely the heterogeneous nucleation during L-PBF.
4. The ductility of the Ti6Al4V-xNb alloy benefits largely from Nb addition. Good ductility (total elongation of 15.6%) and high strength (UTS of 1135 MPa) are obtained with the addition of 10 wt.% Nb (i.e., the Ti6Al4V-10Nb alloy).

The results of this work suggest that L-PBF in situ alloying is a feasible approach to regulating the mechanical properties of Ti alloys. However, it is rather difficult to fully melt the Nb particles, due to the large difference in the melting points between Nb and Ti6Al4V, which may influence the fatigue properties. Future studies are required to optimize the L-PBF in situ alloying process to obtain homogeneous microstructure.

## Data Availability

The raw data supporting the conclusions of this article will be made available by the authors on request.
